# Neural Correlates of Palliative Care: A Feasibility Trial in Patients with Metastatic Gastrointestinal and Lung Cancer

**DOI:** 10.21203/rs.3.rs-9215484/v1

**Published:** 2026-05-11

**Authors:** Eleni Fanouraki, JD Knotts, Cheryl A. Page, Amanda Perry, Tor D. Wager, Thalia P. Wheatley, Christopher Ahern, Amber E. Barnato

**Affiliations:** The Dartmouth Institute for Health Policy and Clinical Practice, Geisel School of Medicine at Dartmouth; Department of Psychological and Brain Sciences, Dartmouth College; Section of Palliative Care, Department of Medicine, Dartmouth Health and Geisel School of Medicine at Dartmouth; The Dartmouth Institute for Health Policy and Clinical Practice, Geisel School of Medicine at Dartmouth; Department of Psychological and Brain Sciences, Dartmouth College; Department of Psychological and Brain Sciences, Dartmouth College; Section of Palliative Care, Department of Medicine, Dartmouth Health and Geisel School of Medicine at Dartmouth; The Dartmouth Institute for Health Policy and Clinical Practice, Geisel School of Medicine at Dartmouth

**Keywords:** palliative care, metastatic cancer, neural correlates, fMRI, end-of-life care, caregiver support

## Abstract

**Background:**

The fundamental affective and decisional processes by which specialty palliative care (SPC) reduces suffering and decreases aggressive treatment at the end of life are not completely understood. We sought to test the feasibility, acceptability, and tolerability of conducting a clinical trial of SPC for advanced cancer patients and their care partners involving functional magnetic resonance imaging (fMRI) to further understand the mechanisms of action of SPC.

**Methods:**

This pilot open-label, single-arm observational case series was conducted at the Dartmouth Cancer Center (DCC) in collaboration with the Dartmouth Brain Imaging Center (DBIC). Participants included patients with metastatic gastrointestinal or lung cancer, their care partners, and their palliative care physician. The intervention consisted of 8–16 weeks of specialty palliative care integrated with usual oncologic care. Assessments included two fMRI study visits involving the collection of physiological measurements during audiovisual stimulation pre- and post-SPC receipt.

**Results:**

The primary outcomes were feasibility (rates of enrollment, intervention receipt), acceptability (closed-ended start and end-of- study feedback for participants and open-ended feedback for non-enrollees), and tolerability (study burden). Of 1216 screened electronic medical records, 21 patients fulfilled the eligibility criteria, of which four patients (19%) and three care partners consented to participate in the study. One participant and their care partner completed all study procedures, one participant withdrew before the first session due to disease progression, and the other two withdrew after 6 and 3 visits, respectively, due to health-related decline, logistical challenges, or emotional concerns.

**Conclusion:**

The study did not achieve its pre-determined criteria for success. Primary reasons for infeasibility were low recruitment and high attrition rates. The study’s results suggest that a more definitive mechanistic study cannot be conducted at this site without addressing critical barriers to enrollment and retention in this vulnerable clinical population.

**Trial registration:**

ClinicalTrials.gov NCT05137782, first release date 11/17/2021

## Introduction

Palliative care plays a critical role in improving quality of life for patients with advanced cancer by addressing the physical, emotional, and existential distress associated with serious illness.^1,2,3^ Patients with metastatic gastrointestinal and lung cancers often experience high symptom burden and complex decision-making needs that also affect their care partners. While evidence supports the clinical benefits of palliative care, including improved mood and quality of life, earlier hospice enrollment, and reductions in burdensome medical treatment at the end of life, the specific psychological and neural mechanisms through which it alleviates suffering remain poorly understood.^4,5^

Recent work has suggested that affective and decisional processes may be central to how palliative care exerts its effects, yet these mechanisms have not been clearly defined.^6,7^ Functional magnetic resonance imaging (fMRI) provides a promising avenue for investigating the neural correlates of these processes. By identifying changes in brain regions related to emotional regulation, stress response, and decision-making, we may be able to clarify how palliative care influences patients' experiences on a biological level.^8,9^ To date, the integration of fMRI into palliative care research has not been explored, particularly among seriously ill patients, where recruitment and retention present major challenges.

To address these gaps, we conducted a single-arm pilot feasibility study to: (1) investigate the neural correlates of specialty palliative care using fMRI, and (2) assess the feasibility, tolerability, and acceptability of the study procedures in preparation for a future larger-scale mechanism-focused trial. By evaluating both the scientific and operational aspects of this study design, we aimed to generate data that could inform more definitive research into the active ingredients of palliative care practice.

The larger, mechanism-focused trial would have compared specialty and primary palliative care for patients with advanced cancer and their care partners. We intended to randomize patients to one of the two models of care and test how these interventions influenced affective and decisional processes, measured through brain imaging, physiological signals, and patient-reported outcomes. Because of the logistical and emotional complexity of conducting fMRI-based research with seriously ill patients, we first evaluated whether recruitment, retention, and study procedures could be carried out using a single-arm design. By piloting these processes in a small sample, we sought to determine whether a two-arm comparative trial could be implemented successfully and to identify procedural barriers that would need to be addressed to scale the study.

## METHODS

### Design and Setting

We conducted a single-arm, open-label pilot feasibility study at the Dartmouth Cancer Center (DCC) at Dartmouth Hitchcock Medical Center (DHMC) in partnership with the Dartmouth Brain Imaging Center at Dartmouth College between January 3, 2023 and November 21, 2023. Dartmouth’s Committee for the Protection of Human Subjects approved the study protocol, study information, and related materials (Protocol No. 02001223). Physicians provided informed consent for participation in the clinical trial. Because this was an open-label design, study assessors were aware of patients’ and surrogates’ assignment to the specialty palliative care intervention.

### Participants

Eligibility for participation was determined through review of patients’ electronic medical records (EMRs). Patients were excluded if they were outside the designated time window since initial metastatic diagnosis, had a cancer diagnosis other than gastrointestinal (GI) or thoracic malignancies, had early-stage disease, presented with brain metastases, were already receiving palliative care or hospice services, were primarily receiving care outside the study site, or had contraindications to functional MRI scanning (e.g., claustrophobia, spinal tumors, or the presence of pacemakers or metal implants). Additional exclusion criteria included poor performance status, limited availability for follow-up, or disease trajectories that required urgent palliative intervention or suggested they were too close to the end of life to complete the study protocol.

### Recruitment

**Figure 1:**
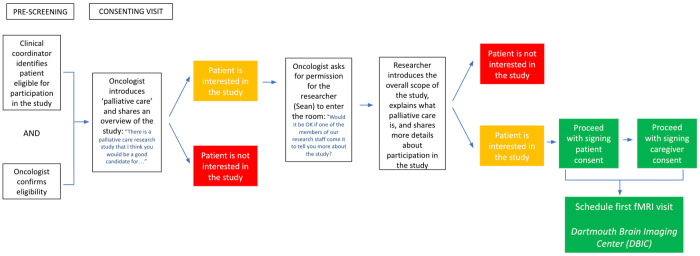
Workflow for Recruitment and Informed Consent Procedures in the Oncology Clinic Setting

Following EMR-based screening, we used a multi-step recruitment process. First, we obtained approval from each patient’s oncologist to ensure there were no clinical or psychosocial factors, not captured in the medical record, that would make it inappropriate to approach the patient. Oncologists introduced the study during routine clinical encounters and assessed patient interest in learning more. If the patient expressed interest, a member of the study team provided a detailed explanation of the research procedures, including the specialty palliative care intervention and fMRI imaging (Appendix I). Written informed consent was obtained during a scheduled oncologic visit, either in the clinic consultation room or in the chemotherapy suite. We told the patient that the study was open to care partners and asked whether they had someone caring for them who would like to participate in the study. We presented the study to care partners if we were introduced. Eligible care partners included family members or informal caregivers involved in the patient’s care outside the hospital setting. For eligible patients who declined participation, brief information regarding reasons for non-participation was documented (Appendix II). Once enrolled, participants (patients +/- their care partners) were scheduled for baseline imaging and introduced to the specialty palliative care team (Figure 1).

### Assessment Visits

All participants received early integrated specialty palliative care (SPC) from a board-certified palliative care physician, while continuing their standard oncologic treatment. SPC visits focused on symptom management, emotional support, and goals-of-care discussions, and occurred at intervals determined collaboratively by the SPC team and the patient. Participants were not randomized and there was no non-SPC comparison arm in this feasibility study. These visits were audio- and video-recorded for analysis of SPC content.

The study assessment visits included two fMRI sessions: one at baseline and one after an 8 to 16-week period of SPC. Participants completed these two in-person study visits at a dedicated fMRI research facility. The purpose of these visits was to examine affective and physiological responses to emotionally evocative stimuli, as well as communication about serious illness, using a multimodal data collection approach.

#### Visit 1 (Baseline)

Visit 1 included a baseline questionnaire, emotion-eliciting tasks conducted while undergoing fMRI, and a recorded initial clinical conversation with an SPC outpatient provider (i.e., an introductory SPC visit including elements of the serious illness conversation guide). Participants first completed a self-administered intake questionnaire assessing psychosocial and emotional factors. While in the scanner, participants viewed 15 short, emotionally evocative film clips (mean ± SD duration = 76.1 ± 23.1 s) with end-of-life themes. At the end of each clip, participants provided ratings on a scale from 1 to 100 about how much the clip made them feel (1) warmth and tenderness, (2) peace, (3) distress, (4) regret or remorse, and (5) like they identified with any characters in the clip. After scanning, participants engaged in their initial palliative care visit in person, which was both video and audio recorded. Discussions (mean ± SD duration = 62.1 ± 9.8 m) were structured to allow observation of real-time affective and physiological responses during a sensitive interpersonal interaction. Continuous skin conductance (palm) and heart rate (thumb) measures were recorded from patients in the fMRI task and from all three conversation participants (patient, care partner, and SPC provider) in the conversation task.^10^ Participants then completed a brief feedback questionnaire.

#### Visit 2 (Follow-up)

**Figure 2:**
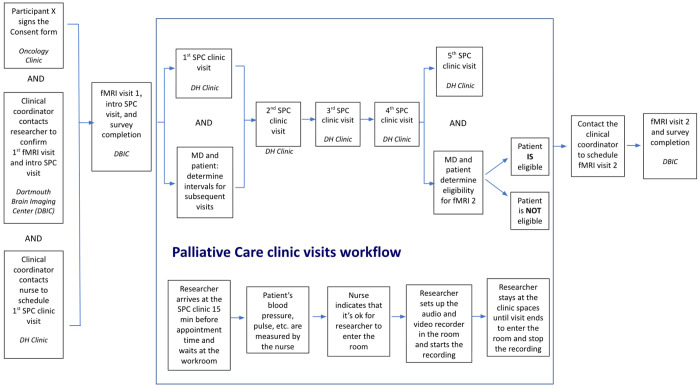
Workflow from Participant Consent to Completion of Second fMRI Session

The second visit was designed to occur approximately 8–16 weeks after Visit 1. One patient and their care partner completed this visit 17 weeks and 2 days following Visit 1. Participants again completed a psychosocial and emotional intake questionnaire prior to scanning. The Visit 1 film clip task was repeated in the scanner, and both participants completed a final feedback questionnaire before the visit concluded (Figure 2).

### Data Collection and Outcomes

Our primary outcomes were feasibility metrics, including rates of recruitment, enrollment, retention, and completion of study procedures. We also assessed tolerability of the imaging procedures and acceptability of the overall study design through structured feedback forms (REDCap surveys) completed by patients and care partners. Secondary outcomes included neural and physiological (heart rate and skin conductance) data during the fMRI sessions and audio-recorded palliative care visits. These preliminary data were intended to inform the feasibility of identifying neural correlates of palliative care exposure in future mechanism-focused trials.

## RESULTS

**Figure 3:**
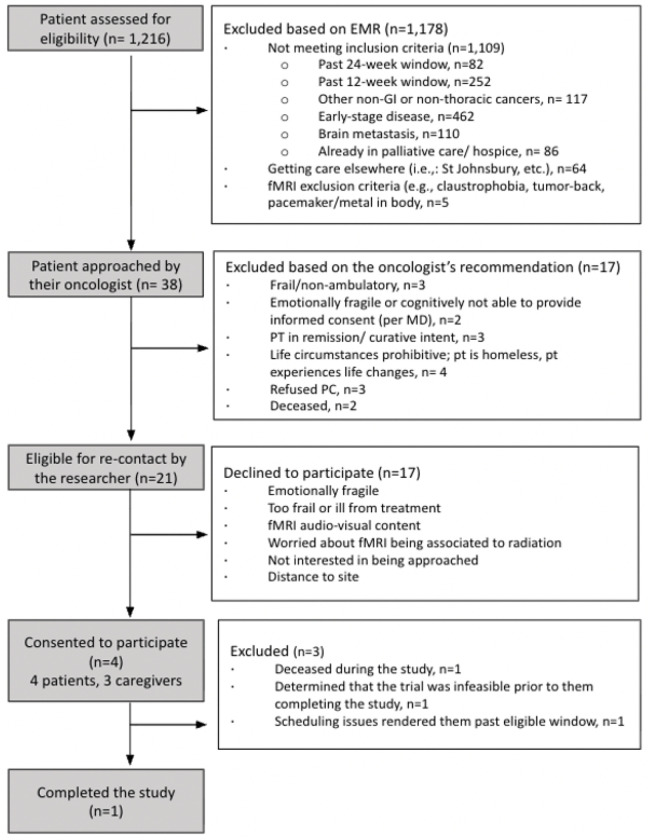
CONSORT Flow Diagram Depicting Participant Screening, Eligibility Assessment, Enrollment, and Study Completion

We screened 1,216 electronic medical records to identify patients with metastatic gastrointestinal or lung cancer. Of these, 21 met the eligibility criteria. In addition, we approached 38 patients through referrals from their oncologists (24 from gastrointestinal oncology and 14 from thoracic oncology) and 17 consented to be contacted by the research team. Ultimately, we enrolled four patients and three care partners. Only one patient-care partner dyad completed the full study protocol, including both fMRI sessions and specialty palliative care visits. One participant withdrew due to disease progression, and the rest did not continue past initial engagement due to health-related decline, logistical challenges, or emotional concerns (Figure 3).

Recruitment rates were significantly lower than anticipated, and attrition rates were high. Despite our structured, multi-step process, beginning with an oncologist’s introduction of the study and followed by detailed discussion and informed consent from the research team, several barriers limited enrollment. Participants commonly cited emotional distress, fear or discomfort related to fMRI, and scheduling conflicts as reasons for declining or discontinuing participation.

Among enrolled participants, adherence to specialty palliative care (SPC) visits was generally high. In one dyad, both the patient and caregiver completed the first fMRI session and all five SPC visits; however, the patient was unable to complete the second fMRI session due to back pain. In a second dyad, the patient completed the first fMRI session and all five SPC visits, while the caregiver participated in SPC visits but did not undergo fMRI scanning due to claustrophobia. A third participant, enrolled without a caregiver, completed the first fMRI session and two of five SPC visits before withdrawing after becoming ineligible when their cancer entered remission. Although preliminary physiological data were collected during fMRI sessions and SPC encounters, the small sample size limited meaningful analysis and interpretation.

Participants who engaged with the study expressed strong support for its aims and consistently indicated willingness to participate in future studies, including under randomized conditions. Feedback reflected high acceptability of the clinician interactions, with participants describing the SPC conversations as comfortable, meaningful, and well conducted. The physiological sensors were reported as unnoticeable or minimally intrusive, and completion of questionnaires was rated as low burden. In contrast, the only reported burden related to the fMRI procedures occurred during one Visit 2 session, when a participant experienced physical discomfort related to underlying back pain (Table 1). These findings suggest that while the intervention components were well received, future studies would benefit from increased flexibility and reduced physical demands, especially for participants managing advanced illness.

Because this was a pilot study, we did not conduct formal power analyses. We conducted a qualitative review of participant feedback to identify common themes around acceptability and study burden. Our results indicate that a larger, mechanism-focused clinical trial comparing primary and specialty palliative care, especially one incorporating fMRI, is not feasible at our current site without major procedural modifications.

## DISCUSSION

This pilot study design was not feasible within the current clinical setting. Despite strong acceptability among enrolled participants, the large screening-to-enrollment ratio and high attrition rates indicate low recruitment efficiency and raise concerns about the scalability of the protocol. These findings suggest that the scientific question remains compelling, but the current protocol requires substantial redesign to be viable in advanced cancer populations.

### Results in context

Prior randomized trials further support the acceptability of SPC interventions among patients with advanced cancer. Early SPC improves psychological outcomes, symptom burden, and quality of life among patients with metastatic lung cancer.^11^ These findings extend to gastrointestinal malignancies, confirming the benefits of integrated SPC across multiple advanced cancer populations, supporting the clinical relevance of studying the “active ingredients” and delivery models of palliative care in this population.^12,13^ Collectively, these studies justify our focus on patients with metastatic gastrointestinal and thoracic cancers, who have demonstrated measurable benefit from SPC interventions.

Our findings extend this literature specifically to the context of high-intensity research among patients with advanced cancer. Completion of study procedures was constrained by disease progression, including high symptom burden, fluctuating functional status, the complexity of coordinating dyadic participation, and logistical challenges such as travel and scheduling around oncology clinic visits. These barriers may be particularly relevant in rural catchment areas, such as our site, and are consistent with the broader oncology literature identifying transportation and access as persistent determinants of both clinical care utilization and research participation.^14^

The low efficiency of our study, even within a structured, clinician-supported recruitment framework, is consistent with existing literature demonstrating that cancer trial participation is frequently limited by structural barriers rather than lack of patient interest alone.^15^ Systematic analyses have shown that structural factors, including eligibility constraints, timing of study introduction, access to research infrastructure, and clinician workflow integration, play a substantial role in limiting participation among otherwise eligible patients.^16^

Clinician-mediated recruitment may have also constrained reach. Clinician engagement and workflow integration are determinants of whether eligible patients are offered participation. Our oncologist-mediated introduction likely increased trust, however, clinician- mediated recruitment can also narrow reach if judgments about “appropriateness to approach” vary across clinicians. Clinicians frequently apply subjective judgments regarding prognosis, emotional readiness, or perceived burden which might limit opportunities for patients who might have otherwise considered participation.^17,18,19^

### Implications

These findings have implications at multiple levels. At the study-design level, they underscore the critical role that feasibility trials play in the design of clinical research studies. While paradigm-changing studies of the neural correlates of chronic pain have seen successful rates of enrollment and completion,^9^ perhaps unsurprisingly, we find that patients burdened not just with pain, but with likely incurable, late stage cancer, are systematically less likely to participate in studies involving time-consuming data collection.

From the participanťs perspective, physical frailty and rapid changes in clinical status present substantial barriers to repeated neuroimaging in advanced cancer populations. However, acceptability can be high despite disease burden being high. Participants reported positive experiences with clinical conversations and minimal burden from sensors and questionnaires, and willingness to consider randomization. This aligns with recent national estimates, which suggest that participation in cancer research is higher than traditionally reported when quality-of-life studies are included, indicating that patients are often willing to engage in research when study opportunities are accessible.^20^

Several design directions follow from our feasibility findings and from prior feasibility work in serious-illness research showing that protocol modifications (e.g., reducing logistical demands and enabling remote components) can improve feasibility and reduce attrition.^21^ Reducing in-person visits, narrowing assessment windows, and incorporating remote or hybrid data collection could improve participation. A more feasible next step in designing a mechanism-based assessment of SPC could involve enrollment of patients without metastatic disease who might nevertheless benefit from the extra layer of support offered by SPC. The disadvantage of this approach is that SPC has not been tested for efficacy in this population, and therefore, assessing the mechanism of action without affirming action would be a potential threat to study validity.

### Limitations

This study has several limitations. Clinician-mediated recruitment, single-site implementation, and the use of an fMRI research facility that was not co-located at the hospital may have introduced selection bias and limited the generalizability of our feasibility study. The intensive nature of the protocol placed substantial demands on research coordinators, which may not be replicable in less well- resourced settings. At the same time, the study has important strengths. The pilot generated empirical evidence about both participantfacing and operational feasibility, including detailed data on screening, enrollment, and data collection. Several elements were successful and should be retained in future work, including clear eligibility criteria, structured recruitment workflows, consent processes tailored to patients with serious illness, creative interdisciplinary collaboration between social psychologists, affective neuroscientists, oncologists, and palliative care physicians, and positive perceptions of the significance and value of the work by enrolled patients and care partners.

## Conclusion

This mechanism-focused trial was not feasible within the current workflow due to low recruitment yield, high attrition, and substantial operational burden. In contrast, participants who engaged viewed the SPC encounters favorably and reported minimal burden from physiologic sensors and questionnaires. Future studies might reduce protocol intensity (i.e., reduce the intensity of neuroimaging requirements or minimize travel and scheduling demands), mitigate clinician gatekeeping effects, or enroll proxy populations (i.e., those without incurable cancer) to enable rigorous mechanistic palliative care research in advanced cancer populations.

## Supplementary Material

This is a list of supplementary files associated with this preprint. Click to download.

• AppendicesMar242026.docx

## Figures and Tables

**Table 1.** Participant Feedback on fMRI Visits and Study Experience

	Patient	Caregiver	Patient	Caregiver	Patient	Caregiver	Patient
Participant ID	S001	S002	S005	S006	S007	S002	S001
**How was the fMRI scan?**	Fine	N/A	N/A	Great	Fine, no problems	N/A	Not great; back pain made it difficult to lie down that long
**Movie clip experience**	I was a little emotional (teary)	N/A	N/A	Emotions were mixed; felt well presented	Felt sad during most of them; sorry for what individuals were experiencing	N/A	N/A
**Physical burden of fMRI scan (0-10)**	0	0	0	0	0	0	8
**Burden of scheduling/getting to fMRI (0-10)**	0	0	0	0	0	0	3
**Conversation with the doctor**	It was a good conversation	[PC clinician’s name] is great! I feel like palliative care should have been introduced to us at our first visit.	As I tend to be uncomfortable in small spaces, I would prefer not to be in a small windowless room. I enjoyed speaking with [PC clinician’s name] and was very comfortable doing so.	The conversation went well. I enjoyed the interaction.	[PC clinician’s name] was great; felt comfortable. Easy conversation.	[Participant’s name] was in too much back pain to complete the scan	N/A
**Experience wearing sensors**	Unnoticeable	Didn’t bother me at all	The sensors were of no concern comfort-wise, but I was more conscious of my arm movement than usual.	Not an issue	Didn’t even notice them	N/A	Comfortable
**Burden of wearing sensors (0-10)**	0	0	0	0	0	0	2
**Burden of questionnaires (0-10)**	0	0	0	0	0	0	3
**Overall study burden (0-10)**	0	0	3	0	0	0	4
**Other bothersome aspects**	None	—	None. The coordinators did a great job.	No	No	No bother at all	Laying down for the MRI
**Would participate if randomized?**	Yes	Yes	Yes	Yes	Yes	Yes	Yes
**Would recommend study?**	Yes	Yes	Yes	Yes	Yes	Yes	Yes

## Data Availability

Data sharing is not applicable to this article as no datasets were generated or analyzed during the current study.
